# Adiponectin in Relation to Coronary Plaque Characteristics on Radiofrequency Intravascular Ultrasound and Cardiovascular Outcome

**DOI:** 10.5935/abc.20180172

**Published:** 2018-09

**Authors:** Bárbara Campos Abreu Marino, Nermina Buljubasic, Martijn Akkerhuis, Jin M. Cheng, Hector M. Garcia-Garcia, Evelyn Regar, Robert-Jan van Geuns, Patrick W. Serruys, Eric Boersma, Isabella Kardys

**Affiliations:** 1 Department of Cardiology, Erasmus MC, Rotterdam - the Netherlands; 2 Universidade Federal de Minas Gerais (UFMG), Belo Horizonte, MG - Brasil; 3 Washington Hospital Center, Washington DC - USA; 4 University Hospital of Zurich, Zurich - Switzerland; 5 Imperial College, London - United Kingdom

**Keywords:** Adiponectin, Atherosclerosis, Plaque, Atherosclerotic, Ultrasonography, Interventional, Coronary Artery Disease / complications.

## Abstract

**Background:**

Prospective data on the associations of adiponectin with in-vivo measurements of degree, phenotype and vulnerability of coronary atherosclerosis are currently lacking.

**Objective:**

To investigate the association of plasma adiponectin with virtual histology intravascular ultrasound (VH-IVUS)-derived measures of atherosclerosis and with major adverse cardiac events (MACE) in patients with established coronary artery disease.

**Methods:**

In 2008-2011, VH-IVUS of a non-culprit non-stenotic coronary segment was performed in 581 patients undergoing coronary angiography for acute coronary syndrome (ACS, n = 318) or stable angina pectoris (SAP, n = 263) from the atherosclerosis-intravascular ultrasound (ATHEROREMO-IVUS) study. Blood was sampled prior to coronary angiography. Coronary plaque burden, tissue composition, high-risk lesions, including VH-IVUS-derived thin-cap fibroatheroma (TCFA), were assessed. All-cause mortality, ACS, unplanned coronary revascularization were registered during a 1-year-follow-up. All statistical tests were two-tailed and p-values < 0.05 were considered statistically significant.

**Results:**

In the full cohort, adiponectin levels were not associated with plaque burden, nor with the various VH-tissue types. In SAP patients, adiponectin levels (median[IQR]: 2.9(1.9-3.9) *µ*g/mL) were positively associated with VH-IVUS derived TCFA lesions, (OR[95%CI]: 1.78[1.06-3.00], p = 0.030), and inversely associated with lesions with minimal luminal area (MLA) ≤ 4.0 mm^2^ (OR[95%CI]: 0.55[0.32-0.92], p = 0.025). In ACS patients, adiponectin levels (median[IQR]: 2.9 [1.8-4.1] *µ*g/mL)were not associated with plaque burden, nor with tissue components. Positive association of adiponectin with death was present in the full cohort (HR[95%CI]: 2.52[1.02-6.23], p = 0.045) and (borderline) in SAP patients (HR[95%CI]: 8.48[0.92-78.0], p = 0.058). In ACS patients, this association lost statistical significance after multivariable adjustment (HR[95%CI]: 1.87[0.67-5.19], p = 0.23).

**Conclusion:**

In the full cohort, adiponectin levels were associated with death but not with VH-IVUS atherosclerosis measures. In SAP patients, adiponectin levels were associated with VH-IVUS-derived TCFA lesions. Altogether, substantial role for adiponectin in plaque vulnerability remains unconfirmed.

## Introduction

Coronary plaque rupture has been described as the main mechanism through which mildly stenotic coronary atherosclerosis can lead to acute coronary thrombosis and myocardial infarction.^1^ High-risk plaques that are vulnerable to such rupture demonstrate distinct morphological characteristics.^2^ They can be differentiated from lesions responsible for stable coronary artery disease (CAD) by their large necrotic cores, thin inflamed fibrous caps, and positive remodeling.^2^ Because plaque vulnerability is associated with inflammation, neovascularization, and necrotic core formation, circulating mediators of these processes may aid in detection of high-risk patients and therefore warrant investigation.^3^ One important inflammatory mediator of CAD is adiponectin. Adiponectin is a protein mainly produced in white adipose tissue, involved in several antioxidant, anti-inflammatory, and anti-atherosclerotic processes.^4-6^ Several studies have demonstrated associations of adiponectin with clinical adverse coronary events.^7-10^ Yet, prospective data on the associations of adiponectin with *in-vivo* measurements of degree, phenotype and vulnerability of coronary atherosclerosis are currently lacking. To further elucidate the pathophysiology of adiponectin in patients with established CAD, we investigated the association of adiponectin with virtual histology intravascular ultrasound (VH-IVUS)-derived measures of degree and composition of coronary atherosclerosis, and with major adverse cardiac events (MACE), in patients undergoing coronary angiography.

## Methods

The design of The European Collaborative Project on Inflammation and Vascular Wall Remodeling in Atherosclerosis - Intravascular Ultrasound (ATHEROREMO-IVUS) study has been described in detail elsewhere.^11,12^ In brief, 581 patients who underwent diagnostic coronary angiography or percutaneous coronary intervention (PCI) for acute coronary syndrome (ACS, n=318) or stable angina pectoris (SAP, n = 263) have been included between 2008 and 2011 in the Erasmus University Medical Center (Erasmus MC), Rotterdam, the Netherlands. The ATHEROREMO-IVUS study was approved by the medical ethics committee of Erasmus MC. The study was performed in accordance with the criteria described in the declaration of Helsinki. Written informed consent was obtained from all included patients. This study is registered in ClinicalTrials.gov, number NCT01789411.

Blood samples for biomarker measurements were drawn from the arterial sheath prior to coronary angiography and were available in 570 patients for the current study. The blood samples were stored at the clinical laboratory of Erasmus MC at a temperature of -80°C within 2 hours after blood collection. C-reactive protein (CRP) was measured in serum samples using an immunoturbidimetric high sensitivity assay (Roche Diagnostics Ltd., Rotkreuz, Switzerland) on the Roche Cobas 8000 modular analyzer platform. These analyses were performed in the clinical laboratory of Erasmus MC. Frozen EDTA plasma samples were transported under controlled conditions (at a temperature of -80°C) to Myriad RBM, Austin, Texas, USA, where adiponectin was measured using a validated multiplex assay (Custom Human Map, Myriad RBM).

Following the standard coronary angiography or PCI procedure, intravascular ultrasound (IVUS) imaging took place in a target segment of a non-culprit coronary artery which was required to be at least 40 mm in length and without significant luminal narrowing (< 50% stenosis) as assessed by on-line angiography. Selection of the non-culprit vessel was predefined in the study protocol. The order of preference for selection of the non-culprit vessel was: (1) left anterior descending (LAD) artery; (2) right coronary artery (RCA); (3) left circumflex (LCX) artery. All IVUS data were acquired with the Volcano s5/s5i Imaging System (Volcano Corp., San Diego, California) using a Volcano Eagle Eye Gold IVUS catheter (20 MHz). An automatic pullback system was used with a standard pullback speed of 0.5 mm per second. The IVUS images were analyzed offline by an independent core laboratory (Cardialysis BV, Rotterdam, the Netherlands) blinded for clinical and biomarker data. The IVUS gray-scale and IVUS radiofrequency analyses, also known as VH-IVUS, were performed using pcVH 2.1 and qVH (Volcano Corp., San Diego, California) software. The external elastic membrane and luminal borders were contoured for each frame (median inter-slice distance, 0.40 mm). Extent and phenotype of the atherosclerotic plaque were assessed. Plaque volume was defined as the total volume of the external elastic membrane occupied by atheroma.^13^ Plaque burden was defined as plaque and media cross-sectional area divided by external elastic membrane cross-sectional area and is presented as a percentage. The composition of the atherosclerotic plaque was characterized into four different tissue types: fibrous, fibrofatty, dense calcium and necrotic core.^14^ A coronary lesion was defined as a segment with a plaque burden of more than 40% in at least three consecutive frames. The following types of VH-IVUS high-risk lesions were identified:

1. thin-cap fibroatheroma (TCFA) lesions: lesions with presence of >10% confluent necrotic core in direct contact with the lumen;^15,16^
2. TCFA lesions with a plaque burden of at least 70%;
3. lesions with a plaque burden of at least 70%;
4. lesions with a minimal luminal area (MLA) of ≤4.0 mm^2^.^11^

Follow-up started at inclusion and lasted 1 year. Post-discharge survival status was obtained from municipal civil registries. Post-discharge rehospitalizations were prospectively assessed during follow-up. Questionnaires focusing on the occurrence of MACE were sent to all living patients. Subsequently, hospital discharge letters were obtained, and treating physicians and institutions were contacted for additional information whenever necessary. ACS was defined as the clinical diagnosis of ST-segment elevation myocardial infarction (STEMI), non-STEMI or unstable angina pectoris in accordance with the guidelines of the European Society of Cardiology.^17-19^ Unplanned coronary revascularization was defined as unplanned repeat PCI or coronary artery bypass grafting (CABG). The primary clinical endpoint was MACE, defined as all-cause mortality, ACS or unplanned coronary revascularization. Secondary endpoints included acute MACE (defined as the composite of all-cause mortality or ACS) and all-cause mortality. The endpoints were adjudicated by a clinical event committee blinded for biomarker and IVUS data.

### Statistical analysis

The distributions of continuous variables, including adiponectin levels and IVUS parameters, were evaluated for normality by visual examination of the histogram. Normally distributed variables are presented as mean ± standard deviation (SD), while non-normally distributed variables are presented as median and interquartile range (IQR). Adiponectin concentration was not normally distributed and was therefore ln-transformed for further analysis. Categorical variables are presented in percentages. We examined associations of adiponectin concentrations with plaque burden, plaque volume, necrotic core fraction, dense calcium fraction, fibro-fatty fraction, and fibrous tissue fraction in the imaged coronary segment by linear regression, with continuous ln-transformed adiponectin concentration as the independent variable. Furthermore, we examined the relation between adiponectin concentrations and the presence of high-risk lesions using logistic regression analyses, with continuous ln-transformed adiponectin concentration as the independent variable. Cox proportional hazards regression analyses were performed to evaluate the relationship between adiponectin concentration and MACE. Clinical variables age, gender, diabetes mellitus, hypertension, and indication for coronary angiography were considered as potential confounders and were entered into the full model. These covariates were a priori chosen based on existing literature, and taking into account the number of events available. CRP was also entered into the full model, as it is the most widely investigated inflammatory marker in CAD, and has been shown to be (inversely) associated with plasma adiponectin levels.^10^ When analyzing the association of adiponectin with the secondary, composite endpoint of death and ACS, and death alone, we only adjusted for age and gender because of the limited number of endpoints.

First, statistical analyses were performed in the full cohort. Then, we included interaction terms (adiponectin multiplied by indication for angiography) into the models to investigate possible effect modification by indication. Subsequently, we repeated the analyses separately in patients with SAP and patients with ACS. All data were analyzed with SPSS software (SPSS 20.0, IBM corp., Armonk, New York). All statistical tests were two-tailed and p-values < 0.05 were considered statistically significant.

## Results

Mean age of the patients was 61.5±11.4 years, 75.4% were men, 17.4% had diabetes mellitus, and median adiponectin concentration was 2.8 (1.9-4.0) *µ*g/mL (Table 1). Coronary angiography or PCI was performed for ACS in 309 (54.2%) patients and for SAP in the remaining 261 (45.8%). Median adiponectin concentration was 2.9 (1.8-4.1) *µ*g/mL in ACS patients and 2.9 (1.9-3.9) *µ*g/mL in SAP patients. A total of 239 (41.9%) patients had at least 1 VH-IVUS-derived TCFA, including 69 (12.1%) patients with at least 1 VH-IVUS-derived TCFA with a plaque burden ≥ 70%.

**Table 1:** Baseline characteristics

	Total (n = 570)	ACS patients (n = 309)	SAP patients (n = 261)
**Patient characteristics**			
Age, years (mean±SD)	61.5 ± 11.4	59.7 ± 11.9	63.6 ± 10.3
Men, n (%)	430 (75.4)	227 (73.5)	203 (77.8)
Diabetes Mellitus, n (%)	99 (17.4)	40 (12.9)	59 (22.6)
Hypertension, n (%)	295 (51.8)	134 (43.4)	161 (61.7)
Hypercholesterolemia, n (%)	317 (55.6)	137 (44.3)	180 (69.0)
Smoking, n (%)	164 (28.8)	115 (37.2)	49 (18.8)
Positive family history, n (%)	293 (51.5)	140 (45.3)	153 (58.6)
Previous MI, n (%)	184 (32.3)	80 (25.9)	104 (58.6)
Previous PCI, n (%)	185 (32.5)	57 (18.4)	128 (49.0)
Previous CABG, n (%)	18 (3.2)	7 (2.3)	11 (4.2)
Previous stroke, n (%)	23 (4.0)	10 (3.2)	13 (5.0)
Peripheral artery disease, n (%)	36 (6.3)	12 (3.9)	24 (9.2)
History of renal insufficiency (%)	32 (5.6)	13 (4.2)	19 (7.3)
History of heart failure, n (%)	19 (3.3)	6 (1.9)	13 (5.0)
**Procedural characteristics**			
*Indication for coronary angiography *			
Acute coronary syndrome, n (%)	309 (54.2)	309 (100)	0 (0)
Myocardial infarction, n (%)	159 (27.9)	159 (51.5)	0 (0)
Unstable angina pectoris, n (%)	150 (26.3)	150 (48.5)	0 (0)
Stable angina pectoris, n (%)	261 (45.8)	0 (0)	261 (100)
*Coronary artery disease*			
No significant stenosis, n (%)	42 (7.4)	18 (5.8)	24 (9.2)
1-vessel disease, n (%)	301 (52.8)	168 (54.4)	133 (51.0)
2-vessel disease, n (%)	166 (29.1)	88 (28.5)	78 (29.9)
3-vessel disease, n (%)	61 (10.7)	35 (11.3)	26 (10.0)
PCI performed, n (%)	501 (87.9)	287 (92.9)	214 (82.0)
**IVUS characteristics**			
Segment length (mm), median (IQR)	44.1 (33.7-55.4)	43.9 [32.9-54.1]	44.8 [34.2-57.2]
Plaque burden (%), median (IQR)	39.2 (30.0-46.4)	37.2 [28.0-45.5]	40.2 [31.8-47.8]
Presence lesion with MLA ≤ 4.0mm^2^	176 (30.9)	88 (28.7)	88 (33.7)
Presence of VH-TCFA, n (%)	239 (41.9)	140 (45.5)	99 (37.9)
Presence of VH-TCFA with PB ≥ 70%, n (%)	69 (12.1)	32 (10.4)	37 (14.2)
**Serum biomarker concentrations**			
C-reactive protein (mg/L), median [IQR]	2.1 [0.8-5.3]	2.8 [1.1-7.0]	1.5[0.6-3.1]
Adiponectin (*µ*g/mL) median [IQR]	2.8 [1.9-4.0]	2.9 [1.8-4.1]	2.9 [1.9-3.9]

ACS: acute coronary syndrome; SAP: stable angina pectoris; SD: standard deviation; MI: myocardial infarction; PCI: percutaneous coronary intervention; CABG: coronary artery bypass grafting; IVUS: intravascular ultrasound; IQR: interquartile range; MLA: minimal luminal area; VH-TCFA: virtual histology thin-cap fibroatheroma; PB: plaque burden.

In the full cohort, adiponectin levels were not associated with composition or burden of atherosclerosis on multivariable analysis (Tables 2 and 3). Adiponectin levels were not associated with MACE after adjustment for age, gender and indication for angiography (Table 4). After further multivariable adjustment, effect estimates remained non-significant (data not shown). Adiponectin levels tended to be univariably associated with acute MACE, (median [IQR] 1.16[0.82-1.62] *µ*g/mL, *vs.* 1.02[0.64-1.38] *µ*g/mL; HR [95%CI]: 1.77[0.96-3.23], p = 0.069), but after further adjustment this tendency disappeared. Adiponectin levels were independently associated with occurrence of death (median[IQR]1.48(1.03-1.79) *µ*g/mL *vs.* 1.02(0.64-1.36) *µ*g/mL, HR[95%CI]: 2.52[1.02-6.23], p = 0.045).

**Table 2:** Association of adiponectin plasma levels with segment intravascular ultrasound characteristics in the total study cohort, acute coronary syndrome and stable angina patients

	IVUS characteristics	Unadjusted Model	p	Multivariable model*	p
		Beta ‡ (95% CI)		Beta ‡ (95% CI)	
All patients (n = 570)	Segment plaque Burden	-0.40 (-2.04 – 1.23)	0.62	-0.95 (-2.74 – 0.85)	0.30
	Dense calcium fraction %	1.35 (0.27 – 2.44)	0.001	0.36 (-0.86 – 1.58)	0.56
	Necrotic core fraction %	0.43 (-0.71 – 1.58)	0.46	0.39 (-0.92 – 1.70)	0.56
	Fibrofatty tissue fraction %	-0.62 (-1.51 – 0.27)	0.17	-0.46 (-1.46 – 0.55)	0.37
	Fibrous tissue fraction %	-1.17 (-2.81 – 0.48)	0.17	-0.29 (-2.16 – 1.58)	0.76
ACS patients (n = 309)	Segment plaque Burden	0.03 (-2.27 – 2.33)	0.98	-0.89 (-3.42 – 1.63)	0.49
	Dense calcium fraction %	2.53 (0.92 – 3.78)	0.001	1.10 (-0.50 – 2.70)	0.18
	Necrotic core fraction %	0.56 (-1.12 – 2.24)	0.51	0.23 (-1.69 – 2.16)	0.81
	Fibrofatty tissue fraction %	-1.47 (-2.78 – -0.15)	0.029	-0.99 (-2.49 – 0.50)	0.19
	Fibrous tissue fraction %	-1.45 (-3.78 – 0.89)	0.22	-0.35 (-3.00 – 2.30)	0.80
SAP patients (n = 261)	Segment plaque Burden	-0.71 (-3.00 – 1.58)	0.54	-0.87 (-3.46 – 1.73)	0.51
	Dense calcium fraction %	0.39 (-1.25 – 2.01)	0.64	-0.41 (-2.28 – 1.47)	0.67
	Necrotic core fraction %	0.24 (-1.29 – 1.77)	0.76	0.57 (-1.20 – 2.35)	0.52
	Fibrofatty tissue fraction %	0.38 (-0.80 – 1.57)	0.52	0.01 (-1.32 – 1.34)	0.99
	Fibrous tissue fraction %	-1.01 (-3.31 – 1.30)	0.39	-0.17 (-2.82 –2.48)	0.90

* Adjusted for age, gender, diabetes, hypertension, and C-reactive protein (CRP). Additionally adjusted for indication for coronary angiography in the total cohort.

† Logarithmically transformed.

‡ Beta per unit increase in ln-transformed adiponectin concentration. IVUS: intravascular ultrasound; CI: confidence interval of 95%; ACS: acute coronary syndrome; SAP: stable angina pectoris; CRP: C-reactive protein.

**Table 3:** Association of adiponectin with presence of virtual histology intravascular ultrasound-derived high-risk lesions in the total cohort, acute coronary syndrome and stable angina patients

		Unadjusted Model	p	Multivariable model*	p
		OR† (95% CI)		OR† (95% CI)	
Total cohort (n = 570)	TCFA	1.11 (0.84 – 1.49)	0.44	1.23 (0.88 – 1.71)	0.23
	TCFA PB ≥70%	0.88 (0.57 – 1.37)	0.55	0.81 (0.50 – 1.33)	0.42
	Lesion with MLA ≤ 4.0 mm^2^	0.84 (0.62 – 1.14)	0.25	0.70 (0.49 – 1.00)	0.052
	Lesion with PB ≥70%	1.02 (0.72 – 1.44)	0.93	0.93 (0.63– 1.39)	0.73
ACS patients (n = 309)	TCFA	0.85 (0.58 – 1.26)	0.42	0.90 (0.58 – 1.42)	0.66
	TCFA PB ≥70%	0.90 (0.57 – 1.42)	0.66	0.77 (0.37 – 1.58)	0.48
	Lesion with MLA ≤ 4.0 mm^2^	1.13 (0.74 – 1.74)	0.57	0.87 (0.53 – 1.44)	0.59
	Lesion with PB ≥70%	1.25 (0.76 – 2.07)	0.38	1.08 (0.60 – 1.94)	0.80
SAP patients (n = 261)	TCFA	1.54 (0.99 – 2.38)	0.057	1.78 (1.06 – 3.00)	0.030
	TCFA PB ≥70%	0.86 (0.48 – 1.52)	0.60	0.87 (0.45 – 1.69)	0.68
	Lesion with MLA ≤ 4.0 mm^2^	0.62 (0.40 – 0.97)	0.035	0.55 (0.32 – 0.93)	0.025
	Lesion with PB ≥70%	0.86 (0.54 – 1.39)	0.54	0.85 (0.49 – 1.47)	0.56

* Adjusted for age, gender, diabetes, hypertension, and C-reactive protein (CRP). Additionally adjusted for indication for coronary angiography in the total cohort. OR: odds ratio; CI: confidence interval of 95%; TCFA: thin-cap fibroatheroma; PB: plaque burden; MLA: minimal luminal area; ACS: acute coronary syndrome; SAP: stable angina pectoris.

† Odds ratio per unit increase in ln-transformed biomarker concentration

**Table 4:** Association of adiponectin with major adverse cardiac events, secondary endpoints and death

		Univariable	p	Adjusted for age and gender†	p
		HR* (95% CI)		HR* (95% CI)	
Total (n = 570)	MACE (n = 56)	1.28 (0.81 – 2.02)	0.29	1.19 (0.71 – 1.99)	0.52
	Acute MACE (n = 32)	1.77 (0.96 – 3.23)	0.069	1.36 (0.68 – 2.72)	0.38
	Death (n = 19)	3.36 (1.49 – 7.59)	0.004	2.52 (1.02 – 6.23)	0.045
ACS (n = 309)	MACE (n = 26)	1.29 (0.66 – 2.50)	0.46	1.02 (0.48 – 2.19)	0.95
	Acute MACE (n = 20)	1.75 (0.81 – 3.72)	0.14	1.40 (0.59 – 3.29)	0.44
	Death (n = 14)	2.44 (0.98 – 6.06)	0.055	1.87 (0.67 – 5.19)	0.23
SAP (n = 261)	MACE (n = 30)	1.30 (0.69 – 2.46)	0.42	1.43 (0.69 – 2.98)	0.34
	Acute MACE (n = 12)	1.75 (0.61 – 4.94)	0.29	1.33 (0.41 – 4.28)	0.64
	Death (n = 5)	8.15 (1.49 – 44.68)	0.016	8.48 (0.92 – 78.03)	0.058

HR: hazard ratio; CI: confidence interval of 95%; MACE: major adverse cardiac events; ACS: acute coronary syndrome; SAP: stable angina pectoris; Acute MACE: composite of death or acute coronary syndrome (secondary endpoints).

† Additionally adjusted for indication for coronary angiography in the total cohort.

* Hazard ratio per unit increase in ln-transformed biomarker concentration

Signs of interactions between adiponectin and indication for angiography were present for associations with TCFA (p for interaction 0.050 (univariable) and 0.029 (multivariable)), with lesions with MLA ≤ 4.0 (p for interaction 0.058 (univariable) and 0.10 (multivariable)), and with fibrofatty tissue fraction (p for interaction 0.042 (univariable) and 0.082 (multivariable)). The remaining interaction terms were not significant (data not shown).

In patients with SAP, adiponectin levels were associated with the presence of VH-IVUS-derived TCFA lesions (median[IQR] 1.16[0.72-1.48] *µ*g/mL *vs*. 0.95[0.62-1.30] *µ*g/mL; OR[95%CI] per 1 unit increase in ln-transformed-adiponectin: 1.78[1.06-3.00], p = 0.030) (Table 3). Furthermore, adiponectin levels were inversely associated with presence of lesions with MLA ≤ 4.0 mm^2^ (median[IQR] 0.95[0.49-1.30] *µ*g/mL *vs.* 1.06[0.69-1.41] *µ*g/mL; OR [95%CI]: 0.55[0.32-0.93], p = 0.025) (Table 3). Finally, adiponectin levels were associated with death (median[IQR] 1.62[1.32-1.84] *µ*g/mL *vs.* 1.02[0.64-1.36] *µ*g/mL; HR [95%CI]: 8.15[1.49-44.68]). After adjustment for age and gender, the HR remained similar in magnitude, although statistical significance was lost (HR [95%CI: 8.48[0.92 - 78.03], p = 0.058).

In patients with ACS, no associations were present between adiponectin and composition or burden of atherosclerosis. Although no associations were present with MACE or acute MACE, a tendency toward a univariable association with death was present (median[IQR] 1.39[0.90-1.86] *µ*g/mL *vs.* 1.01[0.60-1.38] *µ*g/mL; HR [95%CI]: 2.44[0.98-6.06], p = 0.055). After adjustment for age and gender, statistical significance was lost (HR [95%CI]: 1.87[0.67-5.19], p = 0.23).

Given the positive associations we found between adiponectin and death, we performed a post-hoc analysis to explore whether a synergistic effect of adiponectin and TCFA was present on death. For this purpose, we entered interaction terms into the models that consisted of adiponectin multiplied by presence of TCFA lesions. However, no effect modification could be demonstrated (interaction terms were not significant).

## Discussion

To our best knowledge, this is the largest study that correlates circulating adiponectin with *in-vivo* measurements of coronary atherosclerosis using VH-IVUS in patients with known coronary disease. We found that in the full cohort, adiponectin levels were associated with death during 1-year follow-up, but not with VH-IVUS measures of atherosclerosis. In patients with SAP, adiponectin levels were positively associated with presence of VH-IVUS-derived TCFA lesions and were inversely associated with presence of lesions with MLA ≤ 4.0 mm^2^; while the association with death was borderline significant. In ACS patients we only found a tendency toward an association with death during follow-up.

Fundamental experiments, animal models and human studies on vascular function in subjects free of symptomatic cardiovascular disease have all demonstrated associations of adiponectin with vasoprotective mechanisms, including insulin-sensitizing characteristics and anti-oxidative and anti-inflammatory properties.^4-6,8,10^ In line with this, higher levels of adiponectin have been linked to decreased prevalence of CAD in healthy individuals and have demonstrated an inverse association with risk of myocardial infarction.^20,21^ However, in patients with manifested CAD, adiponectin seems to play a different role. When elevated in patients with symptomatic CAD, this adipocytokine becomes associated with an increased risk of cardiovascular events; a phenomenon that has been described under the term "reverse epidemiology".^22-25^ To explain these conflicting findings, it has been proposed that increased adiponectin levels reflect a compensatory and vasculoprotective mechanism.^25^ Specifically, in conditions characterized by a marked systemic pro-inflammatory state and endothelial dysfunction, adiponectin levels increase as an attempt to counter-regulate or compensate for this systemic inflammation. Consequently, the protective effects of adiponectin are superseded by the underlying disease.^25^

In a cohort of 981 patients with stable ischemic heart disease, with average follow-up of 7.1 years, an association was found between higher adiponectin and adverse cardiovascular events (death, heart failure), but after adjustment for cardiac disease severity, the association was no longer statically significant.^24^ Another cohort with median follow-up of 2.5 years found that higher adiponectin levels were associated with future cardiovascular death or nonfatal myocardial infarction in SAP patients (n = 1130), but found no association in ACS patients (n = 760).^22^ Our results, demonstrating an association of adiponectin with death in SAP patients, comply with these findings. The lack of statistical significance for this association in ACS patients in our study may, in part, have been caused by a limited number of clinical events. Moreover, pathophysiological differences may possibly have contributed to the difference we found between SAP and ACS. While in SAP patients, atherosclerosis appears to be a slowly progressing disorder, in ACS patients, coronary plaque rupture may be present, and the latter is accompanied by the production of tissue factor and other homeostatic factors that increase the risk of thrombosis.^3^ Plasma adiponectin levels have been inversely correlated with markers of platelet activation.^26,27^ This might have possibly influenced the association between adiponectin and clinical outcome in these patients.

Adipose tissue produces both pro- and anti-inflammatory adipocytokines,^10^ and adiponectin has shown *in vitro* and *in vivo* anti-inflammatory effects.^28^ However, little is known about the clinical significance of adiponectin for coronary plaque stability *in vivo*. Only a few studies have been performed on this topic, all of which at the University of Kobe, Japan. Sample size of these studies was modest. In a randomized trial of 54 patients with type 2 diabetes and stable angina, treated with pioglitazone, adiponectin was found to be associated with a reduction of necrotic core components as assessed by VH-IVUS.^29^ A case control study of 63 ACS and 43 non-ACS patients showed that serum adiponectin was inversely associated with necrotic core evaluated by VH-IVUS in both culprit and non-culprit lesions in patients with ACS, but not in those with stable angina.^30^ In 50 men with stable CAD, low plasma adiponectin was associated with presence of TCFA.^31^ Altogether, these studies point toward an inverse association of adiponectin with plaque vulnerability. In contrast, in our study, we found a positive association of adiponectin with VH-IVUS TCFA in SAP patients. This finding is in line with the association of adiponectin with death, as well as the association of VH-IVUS TCFA with adverse outcome which we demonstrated earlier.^12^ However, the exact mechanism behind the positive association between adiponectin and VH-IVUS-derived TCFA lesions warrants further investigation. With regard to the discrepancy between our study and the Japanese ones, differences in study population and sample size could have played a part. Ethnic differences in adiponectin levels are of particular interest in this context. The Mediators of Atherosclerosis in South Asians Living in America (MASALA) study and the Multi-Ethnic Study of Atherosclerosis (MESA) have shown that adiponectin levels are lowest in persons from South Asian or Chinese descent compared to other ethnic groups.^32^ Moreover, polymorphisms in the adiponectin gene have been found to be associated with adiponectin levels.^33^ Some of these polymorphisms have also shown associations with insulin resistance, metabolic syndrome and the onset of CAD.^32-34^ Finally, while we found a positive association of adiponectin with VH-IVUS TCFA, we could not demonstrate such an association with necrotic core fraction. This seeming discrepancy may be explained by the fact that these measures reflect somewhat different aspects of atherosclerosis. Size of necrotic core fraction alone may not be able to fully capture the properties of rupture-prone plaques; the definition of VH-IVUS TCFA on its part incorporates additional plaque properties, such as confluence of the necrotic core and direct contact of the necrotic core with the lumen.

Some limitations of this study need to be acknowledged. The spatial resolution of VH-IVUS (200 *µ*m) is insufficient to exactly replicate histopathological definitions of a thin fibrous cap (<65 *µ*m).^13^ Therefore, VH-IVUS tends to over-estimate the number of TCFA lesions. Nevertheless, the presence of VH-IVUS-detected TCFA lesions carries prognostic information^12^ and is therefore clinically relevant. Furthermore, VH-IVUS imaging was performed in a prespecified single target segment of a single non-culprit coronary artery.^35^ This approach was chosen because previous studies have demonstrated that such segments reflect larger coronary disease burden and are associated with subsequent cardiac events.^12,36^ Finally, adiponectin was associated with mortality, but the number of deaths in our dataset was small.

## Conclusion

In conclusion, in the full cohort, adiponectin levels were associated with death but not with VH-IVUS measures of atherosclerosis. In SAP patients, adiponectin levels were associated with VH-IVUS derived TCFA lesions, while the association with death was borderline significant. Altogether, a substantial role for adiponectin in plaque vulnerability remains unconfirmed and warrants investigation by other, large studies.
